# Land Use Shapes Ant Communities: Functional and Compositional Differences Between Oak Forests and Chestnut Orchards in Mediterranean Mountain Landscapes of Northern Portugal

**DOI:** 10.3390/insects17050505

**Published:** 2026-05-15

**Authors:** Camila Lourenço-Lima, Fátima Gonçalves, María Villa

**Affiliations:** 1 CIMO, LA SusTEC, Instituto Politécnico de Bragança, Campus de Santa Apolónia, 5300-253 Bragança, Portugal; camilalima@ipb.pt; 2 Centre for the Research and Technology of Agro-Environmental and Biological Sciences—CITAB, Inov4Agro, Universidade de Trás-os-Montes e Alto Douro—UTAD, Quinta de Prados, 5000-801 Vila Real, Portugal; mariafg@utad.pt

**Keywords:** Formicidae, land use, agroforestry systems, forest ecosystems, natural protected areas, Mediterranean biogeographical region, Atlantic biogeographical region

## Abstract

Ants are widely used as bioindicators because of their sensitivity to environmental change and their functional roles in ecosystems. This study presents the first comparative analysis of ant communities in two habitats, an agricultural system and a semi-natural forest, within the Natural Park of Montesinho (northeastern Portugal). From May to October 2022, four plots were sampled per habitat: (i) semi-natural oak forest and (ii) chestnut orchard under human management, using five pitfall traps in each plot. A total of 1969 ants were captured, representing 32 species and 15 genera. Traditional chestnut orchards supported more exclusive species and greater functional diversity, dominated by generalist and thermophilic taxa. In contrast, oak forests hosted more specialist and cold-adapted species, which may reflect a higher structural stability. Seasonal variation was more pronounced in chestnut orchards, consistent with disturbance-driven dynamics. The functional composition also differed: chestnut orchards favoured granivores and scavengers, while oak forests supported predators and mutualists. These findings highlight the value of ant communities as sensitive indicators of land use and ecosystem condition in Mediterranean mountain systems.

## Simple Summary

Ants are among the most abundant and ecologically important insects in terrestrial ecosystems, where they contribute to soil processes, seed dispersal, and the regulation of other invertebrates. However, little is known about how ant communities respond to different land uses in Mediterranean mountain landscapes of Portugal. In this study, we compared ant communities in two common habitats of a protected area in northern Portugal: traditional chestnut orchards managed for nut production and nearby semi-natural oak forests. Ants were monitored during spring, summer, and autumn to analyze how their communities change across seasons and habitats. Chestnut orchards hosted a higher number of species and genera, likely due to their semi-open structure and diverse ground vegetation. In contrast, oak forests supported species associated with cooler and more stable environmental conditions. For some species, seasonal patterns also differed between habitats, likely reflecting the influence of microclimate on ant activity. These results suggest that traditional chestnut orchards can support diverse ant communities, while oak forests may act as refuges for species adapted to stable forest conditions. Understanding these patterns can help guide landscape management and biodiversity conservation in Mediterranean mountain regions.

## 1. Introduction

Ants (Hymenoptera: Formicidae) are among the most diverse and widespread insect groups, with more than 16,000 species described and many others awaiting discovery [1,2]. Their ecological relevance derives not only from their abundance but also from the multiple roles they play as ecosystem engineers, influencing soil structure, nutrient cycling, and seed dispersal [2,3,4].

Because ant communities respond sensitively to environmental variation and land-use change, they have been widely used as bioindicators in ecological monitoring and restoration [5,6,7,8]. Under increasing land-use intensification, declines in local species richness have frequently been reported. This negative trend has been documented in grasslands, cocoa plantations, forest–savanna transition landscapes, and savannas converted to cropland, where less intensively managed sites generally support higher species richness than more intensively managed agricultural systems [9,10,11].

Despite their ecological importance and recognized utility as bioindicators, in Portugal, the diversity and functional structure of ant communities—particularly within natural protected areas—remain poorly documented. This contrasts with broader arthropod surveys (e.g., [12]) or better-studied taxa such as spiders (e.g., [13]). Although regional assessments have been conducted in Spain for protected areas and large river basins (e.g., [14,15]), research in continental Portugal is still largely limited to a few localized studies (e.g., [16,17,18,19,20]). To date, approximately 138 ant species have been reported for continental Portugal [21,22,23]. In northern Portugal specifically, ecological studies focusing on ants are scarce and mostly restricted to agricultural systems, such as vineyards [24] and olive groves [25].

The Natural Park of Montesinho (NPM), located in northeast Portugal, is one of the largest protected areas in the country. Its landscape reflects long-term traditional land use and forms a heterogeneous mosaic of hills and river basins across a marked altitudinal gradient (438–1481 m) [26]. The mountainous terrain accentuates climatic gradients, where orographic effects generate contrasts in precipitation and temperature. This environmental heterogeneity, coupled with its geological and geomorphological complexity, supports a rich diversity of plant communities and habitat types [27]. Two habitats are particularly representative of the NPM: semi-natural oak forests (*Quercus rotundifolia* Lam.) and chestnut orchards (*Castanea sativa* Mill.). Oak forests, traditionally managed through low-intensity practices such as firewood collection and understory clearing, are characterized by slow-growing, drought-resistant (xerophytic) tree species that promote relatively stable microclimatic conditions and complex trophic networks, acting as refuges for biodiversity [28,29]. In contrast, chestnut orchards are cultivated mainly for nut production and therefore receive a higher level of human management than oak forests (e.g., pruning and harvesting). Their more open canopies allow a highly biodiverse herbaceous vegetation, which in the region is managed under a wide range of practices, from soil tillage and herbicide application to mowing prior to harvest. Despite ongoing pressures from rural depopulation, land-use change, and phytosanitary threats such as the chestnut gall wasp *Dryocosmus kuriphilus* Yasumatsu (Hymenoptera: Cynipidae), chestnut orchards remain ecological and cultural elements of the region [30,31].

Consistent with the broader national pattern, the myrmecofauna of these habitats within the NPM remains largely uncharacterized. Previous research in the NPM has more frequently targeted charismatic or taxonomically attractive groups such as Lepidoptera, Orthoptera, and Odonata [32,33,34]. In chestnut ecosystems studies have predominantly addressed pest species or biological control [35,36]. Addressing this knowledge gap is essential to evaluate habitat quality, understand seasonal patterns of activity, and assess the potential role of ants as bioindicators in Mediterranean mountain ecosystems.

Within this context, the present study focuses on two contrasting and representative habitat types—semi-natural oak forests and traditionally managed chestnut orchards. Specifically, this study aims to: (i) characterize and compare the diversity, composition, and functional structure of ant communities in oak forests and chestnut orchards in representative sites of the NPM; (ii) assess the seasonal variation of the ant community structure; and (iii) evaluate the potential of ants as indicators of ecological integrity and land-use impacts in this protected area.

## 2. Materials and Methods

### 2.1. Study Sites

**Figure 1:**
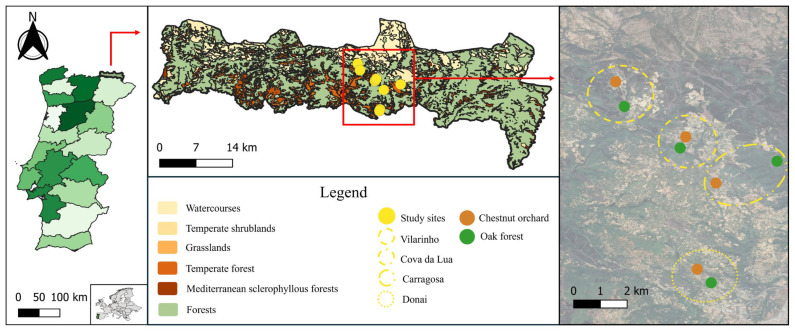
Map of Portugal (left); the location of the study areas within the NPM (middle); the location of the sample sites within the study area (right) (image obtained from Google Earth^®^).

Four study areas in the central part of NPM (Figure 1) were selected, each located in different villages: Vilarinho, Cova da Lua, Carragosa, and Donai. In each area, one habitat with human intervention (chestnut orchard) and one semi-natural habitat (oak forest) were sampled, totaling four chestnut orchards and four oak forests.

The study plots were embedded within heterogeneous landscape matrices. Oak forest plots were generally surrounded by a mosaic of similar oak stands and grasslands, and to a lesser extent by chestnut orchards, riparian vegetation, and other woodland types, predominantly *Quercus pyrenaica* stands. Chestnut orchard plots were primarily located within agricultural matrices dominated by other chestnut orchards, interspersed with grasslands, shrublands, and patches of oak forest. Altitude ranged from 803 to 937 m across chestnut orchards and from 805 to 867 m in oak forests. The distances between the study areas ranged from 2 to 5 km. The distance between paired habitats within areas ranged from 20 m to 1 km. Plot sizes ranged from 2000 to 7000 m^2^ for chestnut orchards and 6700 to 8400 m^2^ for oak forests. The climate is Mediterranean with continental influence, characterized by cold winters and hot summers, with mean annual temperatures around 10–12 °C and annual precipitation ranging between 800 and 1200 mm [27]. Climatic conditions during the study period are described in the Supplementary Materials.

### 2.2. Ant Sampling

Ants were collected from May to October 2022, once per month, using pitfall traps. The pitfall traps consisted of plastic cups with a depth of 16 cm and a diameter of 9 cm (MAC220T-0018, PBP, Net Comerce Lda, Santa Iria de Azoia, Portugal) buried in the soil with a minimum distance of 25 metres between them—to minimize spatial dependence among sampling units—and filled with 150 mL of a solution of water and polypropylene-glycol (3:1) and four drops of detergent to reduce surface tension and facilitate submersion of ants. To prevent the capture of small mammals and minimize excessive rainwater entry, each trap was covered with a plastic lid, supported by three wires inserted into the ground, creating a 2 cm gap between the lid and the soil. This method has been widely employed in numerous studies of ant communities globally (e.g., [37]), as it is a standard, cost-effective approach for collecting epigean ants, yielding robust data on species richness and composition patterns while facilitating continuous day and night sampling [38].

**Figure 2:**
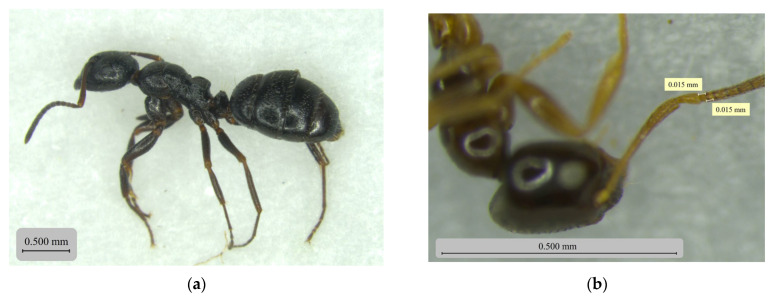

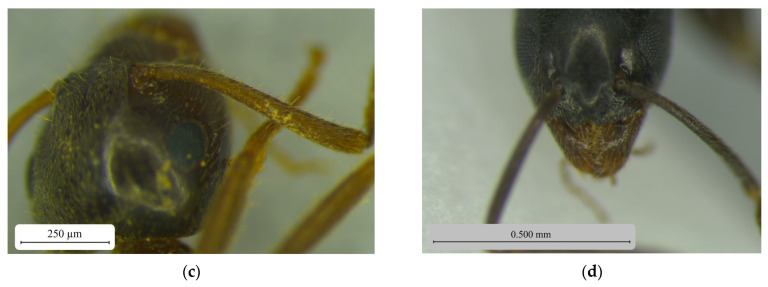
Detailed views of four representative species obtained under a stereomicroscope: (**a**) Lateral view of a specimen of *Camponotus piceus*; (**b**) Dorsal view of the antennal detail of *Plagiolepis schmitzii*; (**c**) Posterior view of the head and antennal detail of *Lasius grandis*; (**d**) Frontal view of the head of *Tapinoma erraticum*, detailing the clypeal emargination.

In each study plot, five pitfall traps were randomly distributed, with a minimum distance of 25 m between traps and between each trap and the plot boundary. This resulted in 20 traps per habitat and 40 traps per sampling date. The same sampling points were maintained throughout sampling dates. Samples were collected after seven days. In the laboratory, the traps were filtered, and the ants were preserved in 70% alcohol until sorting and identification. Subsequently, specimens were separated and identified to the species level. Taxonomic identification was performed with a stereomicroscope (Leica MicroSystems Microscopes, EZ4 Fixed 10x 10447197, Maia, Portugal), according to morphometric measurements (Figure 2), specialized guides, such as “Hormigas de La Península Ibérica e Islas Baleares” [23] and cross-comparisons with high-resolution images of type specimens available on the AntWeb database [39].

### 2.3. Data Analysis

#### 2.3.1. Ant Community

A Venn diagram was constructed to visualize the number of exclusive and shared species in the environment using the venn.diagram function from the VennDiagram package [40] in R 4.4.1 [41]. Community similarity between environments was quantified using the Sørensen–Dice index, calculated both for the entire study period and separately for each sampling month, according to the following formula:

$$Sørensen-Dice\;index=\frac{2\times total\;Species}{Species\;in\;chesnut+Species\;in\;oak\;forest}$$

To analyze variation in ant community composition, we performed a Non-metric Multidimensional Scaling (NMDS) ordination based on Bray–Curtis dissimilarity. Ant species were divided into two groups according to their total abundance across all sampling plots and months: (i) abundant species, with more than 40 individuals recorded (n = 10), and (ii) rare species, with 40 or fewer individuals (n = 25). This separation was applied to reduce the influence of a few numerically dominant taxa and to allow community patterns to be assessed for both dominant and less frequent species. The threshold (>40 individuals) corresponds to a marked change in slope in the rank–abundance curve (Figure S1, Supplementary Materials), separating a small set of dominant species from the long tail of low-abundance species. Rare species were retained in the analysis because they may represent habitat specialists or indicators of specific microhabitats.

Prior to ordination, abundance data were log(x + 1) transformed to reduce the influence of highly dominant species. NMDS ordination was performed in two dimensions (k = 2) using the metaMDS() function from the vegan package 2.6.6.1 [42], with trymax = 100 and set.seed(123) to ensure reproducibility. Species vectors were fitted to the ordination space using the envfit() function with 999 permutations.

Differences in species composition among environments and sampling months were tested using Permutational Multivariate Analysis of Variance (PERMANOVA) based on Bray–Curtis dissimilarities with 999 permutations, implemented with the adonis2() function from the vegan package. Analyses were conducted separately for abundant and rare species.

To verify that PERMANOVA results were not driven by differences in multivariate dispersion rather than by differences in community composition, the homogeneity of multivariate dispersion was tested using the betadisper() function from the vegan package, also based on Bray–Curtis dissimilarities. Statistical significance was assessed using ANOVA.

#### 2.3.2. Species-Level Response to Habitat and Month

To assess the response of the most abundant species to habitat and seasonal variation, a series of Generalized Linear Models (GLMs) were fitted. In these models, species abundance was used as the response variable, and habitat (chestnut orchard and oak forest), sampling month (May, June, July, September, and October), and their interaction as explanatory variables. Plot or study area were not included as factors in the models due to the limited number of replicates at that level, which prevented robust estimation of their effects. This should be considered a limitation of the study design.

To moderate the influence of extremely high captures, potentially due to multiple individuals from a single nest being caught in the same pitfall trap, Winsorization was applied. Specifically, values above the upper limit (Q3 + 1.5 × IQR) were truncated to that threshold, where Q is the quartile and IQR is the interquartile range.

The Negative Binomial distribution for overdispersed count data was used. Different model structures were explored to describe potential seasonal patterns, including linear and non-linear seasonal patterns. Non-linear seasonal patterns were modelled using second-degree polynomials (allowing parabolic responses), third-degree polynomials (allowing more complex seasonal curves with up to two inflection points), and natural splines with four degrees of freedom, which provide flexible smooth responses without the instability associated with higher-order polynomials. Zero-inflated models were also tested to account for excess zeros. Competing models were compared using Akaike’s Information Criterion (AIC), and the model with the lowest AIC was selected as the final model. Models were fitted using the glm.nb function from the package MASS 7.3.60.2 [43] and glmmTMB function from glmmTMB package 1.1.9 [44]. Model validation was conducted using residual diagnostics from the simulateResiduals() function in the DHARMa package 0.4.6 [45].

#### 2.3.3. Community-Level Response to Habitat and Month

Ant richness and Shannon diversity were modelled using Generalized Additive Models (GAMs). Explanatory variables included habitat and sampling with month modelled as a smooth term to capture potential non-linear seasonal patterns, as well as their interaction. Shannon diversity was modelled assuming a Gaussian distribution, whereas richness was modelled using a negative binomial distribution to account for overdispersion in count data. Models were fitted using the gam function from the mgcv package 1.9.3 [46]. Model validation and selection were performed as described in the previous section.

## 3. Results

### 3.1. Ant Community

**Figure 3:**
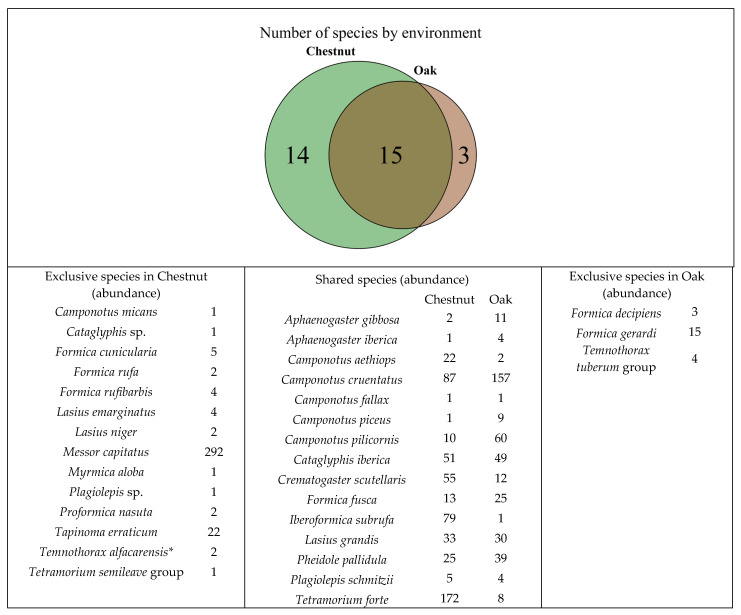
Venn diagram showing the number of ant species exclusive to and shared by the environment (chestnut and oak forest). Species and number of captured specimens by environment are shown. * *Temnothorax alfacarensis* is addressed in detail in a separate contribution (manuscript under review).

In total, 1969 ants were captured, with 1377 individuals in the chestnut orchard and 592 in the oak forest. The ant community comprised 15 genera and 32 species. Species belonged to the subfamilies Formicinae (18 species), Myrmicinae (10), and Dolichoderinae (1). Among the genera found, *Camponotus* (6 species) was the most represented, followed by *Formica* (5 species), *Lasius* (3), and *Tetramorium* (3). Fifteen species were shared by both habitats, 14 were exclusive to chestnut orchards, and three were exclusive to oak forests (Figure 3).

The three most abundant species overall were *Messor capitatus* (Latreille, 1798), *Camponotus cruentatus* (Latreille, 1802), and *Tetramorium forte* (Forel, 1904). However, the abundance of *M. capitatus* was skewed by a single sample from a chestnut orchard, which contained 547 individuals, representing 78% of all individuals recorded for that species. In contrast, *C. cruentatus* and *T. forte* showed a more consistent distribution, with an average of 10 individuals in samples where they were recorded. Ant activity, measured by abundance, peaked in both habitats during June and July. This period coincided with evidence of nuptial flights in the chestnut orchard, confirmed by the capture of two-winged males and four queens.

Ant community composition showed clear differences between habitats. In the chestnut orchard, species associated with warm and dry climates were dominant, accounting for 78.4% of individuals, with a higher incidence in June and July. In the oak forest, although warm-climate species also predominated (59%), a higher incidence of cold-climate species (e.g., *Camponotus pilicornis* (Roger, 1859)) was observed, constituting 12% of the individuals, compared to only 1% in the chestnut orchard. Species with neutral climatic preferences were most active in July and September (Table 1).

Regarding feeding habits (Table 1), granivores, scavengers, generalists, and insectivores were more abundant in the chestnut orchard. In contrast, predators and aphid-tending species were more abundant in the oak forest. Nectivorous species were abundant in both habitats, although their activity peak in chestnut orchards occurred from May to July, and in oak forests from June to September.

The Sørensen–Dice similarity index between chestnut orchards and oak forests across the entire sampling period was 0.625. Similarity varied across months, with the lowest value in June (0.118), intermediate values in May (0.381) and October (0.400), and the highest values in July (0.611) and September (0.583). Overall, similarity was higher in summer than in spring or autumn.

**Figure 4:**
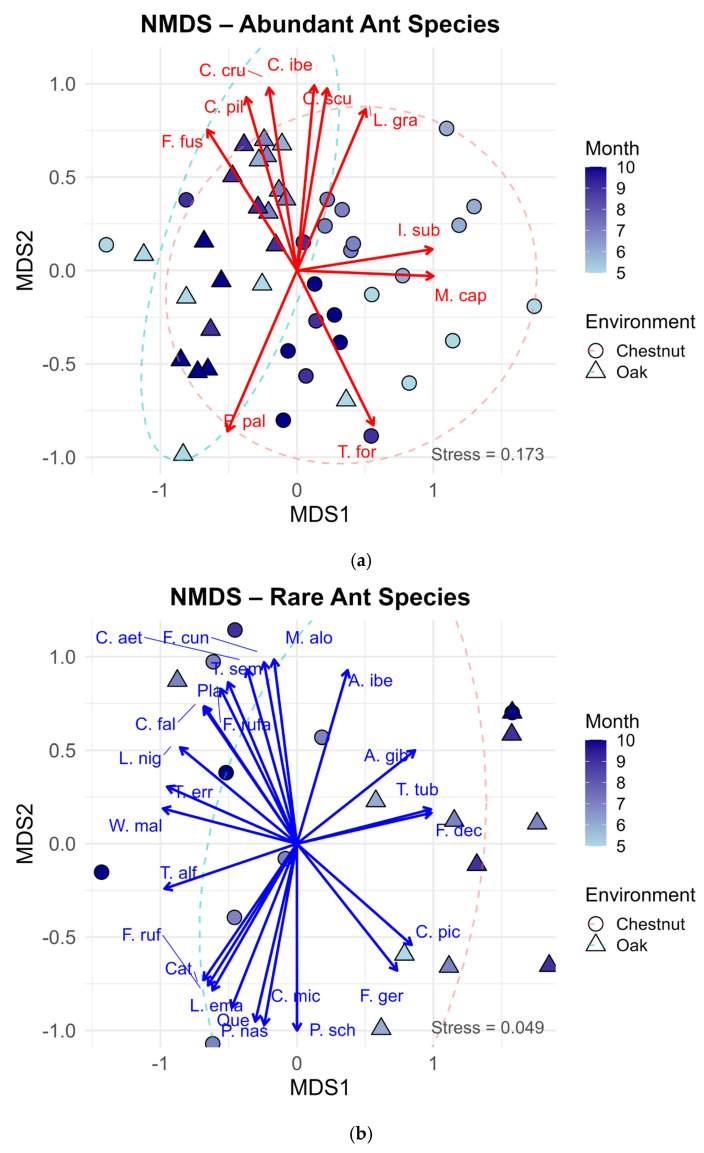
Non-metric multidimensional scaling (NMDS) ordination plots based on Bray–Curtis dissimilarity of ant communities. (**a**) Abundant species (>40 individuals total); (**b**) Rare species (≤40 individuals total). Points represent samples, filled by month and shaped by environment (chestnut orchard vs. oak forest). Ellipses indicate 95% confidence intervals around environments. Arrows indicate the direction and strength of species most strongly associated with the ordination axes. NMDS stress values indicate an acceptable fit for abundant species (<0.2) and a good fit for rare species (stress < 0.1). Abbreviations: *Aphaenogaster gibbosa*—A. gib, *A. iberica*—A. ibe, *Camponotus aethiops*—C. aet, *C. cruentatus*—C. cru, *C. fallax*—C. fal, *C. micans*—C. mic, *C. piceus*—C. pic, *C. pilicornis*—C. pil, *Cataglyphis*—Cat, *C. iberica*—C. ibe, *Crematogaster scutellaris*—C. scu, *Formica cunicularia*—F. cun, *F. decipiens*—F. dec, *F. fusca*—F. fus, *F. rufa*—F. rufa, *F. rufibarbis*—F. ruf, *F. gerardi*—F. ger, *Iberoformica subrufa*—I. sub, *Lasius emarginatus*—L. ema, *L. grandis*—L. gra, *L. niger*—L. nig, *Messor capitatus*—M. cap, *Myrmica aloba*—M. alo, *Pheidole pallidula*—P. pal, *Plagiolepis*—Pla, *Plagiolepis schmitzii*—P. sch, *Proformica nasuta*—P. nas, *Tapinoma erraticum*—T. err, *Temnothorax alfacarensis*—T. alf, *T. tuberum* group—T. tub, *Tetramorium forte*—T. for, *T. semileave* group—T. sem, Queen—Que, Winged male—W. mal.

The NMDS ordination illustrated clear patterns in ant community composition. For abundant species (Figure 4a), samples clustered distinctly by both month and habitat, suggesting that community structure is driven by temporal and environmental factors. Notably, samples from chestnut orchards in the summer months (June and July) clustered distinctly from spring and autumn samples and from oak forest samples, suggesting differences in community structures. This separation was further supported by the non-overlapping confidence ellipses for the two habitats. Species vectors indicated that taxa such as *M. capitatus* and *Iberoformica subrufa* (Roger, 1859) were strongly associated with chestnut orchards in the summer, while *T. forte* was linked to the same habitat in autumn. In contrast, species like *Formica fusca* (Linnaeus 1758), *C. pilicornis*, and *C. cruentatus* were more closely associated with oak forests.

For rare species (Figure 4b), the ordination revealed a more diffuse pattern, reflecting greater stochasticity or microhabitat specialization among these taxa. However, certain months or environments still showed localized clustering, and species vectors suggest that some rare species were associated with specific conditions, possibly acting as indicators of particular microhabitats, such as *Aphaenogaster gibbosa* (Latreille, 1798), *Formica decipiens* (Bondroit, 1918) and *Camponotus piceus* (Leach, 1825) that seemed to be associated with oak forest in the later summer or *Tapinoma erraticum* (Latreille, 1798), related to chestnut orchard.

For abundant species, the PERMANOVA indicated significant differences in community composition between environments (R^2^ = 0.179, *p* = 0.001), confirming the separation observed in the NMDS ordination. Both environment and month had significant effects (Environment: R^2^ = 0.179, *p* = 0.001; Month: R^2^ = 0.082, *p* = 0.001). No significant interaction was found between environment and month, so it was excluded from the final model.

For rare species, PERMANOVA also revealed a significant effect of environment on community composition (R^2^ = 0.118, *p* < 0.001), although the explanatory power was slightly lower compared to abundant species. No significant effect of month was detected for rare species.

The test of homogeneity of multivariate dispersions (PERMDISP) indicated no significant differences in beta diversity dispersion between environments for abundant or rare species (abundant: F_1_,_44_ = 1.38, *p* = 0.247; rare: F_1_,_31_ = 1.72, *p* = 0.199). This suggests that the significant PERMANOVA results are likely due to differences in community composition rather than differences in variability among groups.

### 3.2. Species-Level Response to Habitat and Month

**Figure 5:**
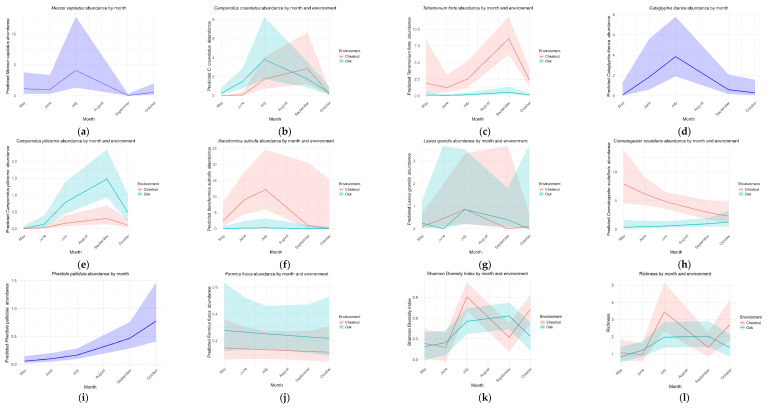
Plots for the models fitted for the response of the abundance of *Messor capitatus* (**a**), *Camponotus cruentatus* (**b**), *Tetramorium forte* (**c**), *Cataglyphis iberica* (**d**), *Camponotus pilicornis* (**e**), *Iberoformica subrufa* (**f**), *Lasius grandis* (**g**), *Crematogaster scutellaris* (**h**), *Pheidole pallidula* (**i**), *Formica fusca* (**j**), ant Shannon Diversity Index (**k**), Richness (**l**).

The response of each species to habitat and month varied among taxa (Figure 5 and Table S1).

*Messor capitatus* showed a nonlinear seasonal pattern, with lower abundance towards autumn. *C. cruentatus* exhibited a marked seasonal pattern with a peak in July in oak forests, while abundance in chestnut orchards increased later in the season. Overall, the species was significantly more abundant in oak forests, with a significant interaction between month and habitat. *T. forte* also showed significant seasonal variation and a strong habitat effect, being less abundant in oak forests than in chestnut orchards. *Cataglyphis iberica* (Emery, 1906) displayed a pronounced seasonal peak in July, while *C. pilicornis* exhibited a clear seasonal pattern and was more abundant in oak forests. *I. subrufa* showed a non-linear seasonal trend, with lower abundance in oak forests. In contrast, *L. grandis* showed only a marginal seasonal effect and no significant influence of habitat. *Crematogaster scutellaris* (Olivier, 1792) responded significantly to month, habitat, and their interaction, with abundance decreasing over time, particularly in chestnut orchards. *Pheidole pallidula* (Nylander, 1849) showed significant seasonal variation, with abundance increasing towards autumn. Finally, for *F. fusca*, neither month nor habitat had a significant effect on abundance.

### 3.3. Community-Level Response to Habitat and Month

Regarding the Shannon Diversity Index and ant species richness, the GAMs showed that while mean diversity did not differ significantly between oak and chestnut forests, strong seasonal trends were observed in both environments. These patterns were stronger in chestnut orchards than in oak forests, indicating that richness varied markedly across months depending on habitat (Figure 5, Table S1).

## 4. Discussion

This research presents the first comparative analysis of ant communities across relevant habitats in the Natural Park of Montesinho, revealing clear differences between agricultural chestnut orchards and semi-natural oak forests.

### 4.1. Taxonomic Composition and Diversity

While agricultural intensification is often associated with biodiversity loss [47,48,49,50], previous studies have shown that traditional or low-intensity systems, including secondary forests and woody agroforestry systems, can support high levels of ant diversity [11]. In our study, traditional chestnut orchards hosted a higher number of exclusive taxa than semi-natural oak forests. This highlights the context-dependent nature of agricultural impacts, as low-intensity systems can maintain or even enhance certain components of biodiversity. Moreover, a higher species diversity of the genus *Lasius* (e.g., *L. grandis*, *L. emarginatus*, *L. niger*) was observed in the chestnut orchard, together with the exclusive presence of genera such as *Myrmica*, *Proformica*, and *Temnothorax*.

This pattern suggests that the semi-open structure of chestnut orchards may provide greater microhabitat heterogeneity potentially including areas of exposed soil, varying levels of solar exposure, and a more diverse herbaceous vegetation layer, which are likely less available in more homogeneous and closed-canopy habitats such as oak forests. Under this hypothesis, increased microhabitat heterogeneity may support a richer ant fauna composed of both forest and open-area species [51], in accordance with the habitat heterogeneity hypothesis, which predicts positive effects of structural complexity on species coexistence [52].

In addition, this pattern is also consistent with the intermediate disturbance hypothesis, which predicts that habitats experiencing moderate levels of disturbance may support higher diversity by allowing the coexistence of both disturbance-tolerant and more competitive species [53]. Despite the higher number of taxa recorded in chestnut orchards, these communities were largely dominated by disturbance-tolerant generalist species. In contrast, oak forests supported a greater relative importance of species associated with cooler and more stable microclimatic conditions. This suggests that land use may act as an environmental filter: disturbance and management in chestnut orchards would favour generalist and opportunistic species adapted to warm and dry conditions, while the more stable and shaded conditions of oak forests would allow the persistence of species associated with cooler microclimates.

Similar patterns have been reported in other Mediterranean ecosystems, where disturbance tends to promote generalist species and reduce the occurrence of habitat specialists [9,10].

However, these results should be interpreted with caution. Ant community composition and species diversity may vary between years in response to interannual climatic variability, particularly fluctuations in temperature, which can strongly influence ant activity and detectability. In this context, some of the low-abundance species recorded and classified as habitat-specific may not be strictly exclusive to a given habitat, but may partly reflect sampling limitations or temporal variability. Therefore, longer-term studies would be necessary to confirm the consistency of these patterns and to better assess the stability of habitat-associated species across years.

### 4.2. Seasonal Dynamics

The temporal dynamics of diversity are consistent with this interpretation. The Sørensen–Dice similarity index between habitats was moderate overall but varied seasonally, reaching minimum values in June and maximum values in July, indicating that the communities were most similar at the peak of summer. The peak in similarity during July may reflect a period of maximum ant activity, when species from both habitats are simultaneously active and therefore more likely to be detected, as ant foraging activity is strongly influenced by temperature and seasonal climatic conditions [2]. The chestnut orchard exhibited marked seasonal fluctuations in species richness, with a peak in summer followed by a decline in autumn. Such variability is expected in more open habitats, where stronger microclimatic fluctuations influence ant activity and community composition. In contrast, oak forest richness remained remarkably stable, reflecting the more buffered microclimatic conditions typical of closed-canopy systems. This pattern is consistent with disturbance-mediated increases in habitat openness favouring generalist species while disadvantaging closed-habitat specialists [54]. Moreover, some taxa responded differently to seasonal conditions depending on habitat. For example, *C. cruentatus* showed earlier activity peaks in oak forests than in chestnut orchards, suggesting that microclimatic differences between habitats may influence the timing of foraging activity.

### 4.3. Functional Structure and Ecological Implications

The contrast is also visible in the functional structure of the communities. Chestnut orchards were dominated by guilds that exploit ephemeral and abundant resources, such as granivores, scavengers, and nectivores. Their open canopy and non-tillage management may promote the development of herbaceous vegetation, which could increase seed and nectar supply. For example, the granivore *M. capitatus*, which possesses a robust head and powerful mandibles adapted for seed transport [55], peaked in summer, coinciding with the period of maximum seed availability [56]. A similar pattern was observed in *T. forte* during autumn. As a ruderal or pioneer species, it is well adapted to habitats subject to recurrent disturbance, such as agroecosystems [57], where its feeding plasticity allows it to exploit temporary food resources exposed during harvest.

The oak forest community included more species with specialized strategies, such as aphid-tending and predation, which may be associated with more stable environmental conditions. Ant–aphid mutualisms involve energetic costs for both partners: aphids invest in producing honeydew while ants allocate time and effort to its collection and defence [58]. The perennial tree layer of oak forests may provide relatively stable substrates and persistent plant resources that may facilitate these interactions. In contrast, the higher disturbance associated with agricultural management in chestnut orchards may limit the persistence of such interactions through time.

The importance of predators in the oak forest may indicate greater trophic complexity under less disturbed conditions. Species such as *C. pilicornis*, which was significantly more abundant in oak forests, and *F. fusca*, are associated with structural features typical of mature forest ecosystems. *F. fusca*, for example, often nests in deadwood, a resource scarce in managed forests but characteristic of older forest stands [59]. Species that depend on deadwood are therefore widely considered indicators of ecological continuity [60]. Similarly, many species of the genus *Camponotus* are associated with mature trees and the complex microhabitats they provide, making them reliable indicators of structurally complex and stable forest environments [54].

Although *C. iberica* and *P. pallidula* showed similar total abundances in both habitats, their activity phenologies differed markedly. *C. iberica* exhibited a peak in abundance during the summer, consistent with the thermophily and adaptations of this genus for foraging at high temperatures, including in arid environments [61,62]. In contrast, *P. pallidula* showed a progressive increase in abundance from May to October. Although disturbance in chestnut orchards could be expected to favour this generalist species, no significant difference was observed between habitats. This pattern may suggest that its activity cycle is primarily driven by seasonal temperature rather than habitat conditions. Additionally, as a “super-dominant” species in Mediterranean ecosystems, *P. pallidula* may reach high population densities across a wide range of suitable habitats [63].

An antagonistic pattern was observed for *C. scutellaris*, whose abundance decreased in the chestnut orchard while increasing in the oak forest. This pattern may be related to the species’ polydomous nest structure and the seasonal relocation of satellite nests. *C. scutellaris* is known to maintain a system of multiple, interconnected nests, which provides enormous flexibility in resource exploitation [64]. As a primarily arboreal species [65], it is plausible that the colony abandons satellite nests in the chestnut trees as they become unfavourable with leaf fall in autumn. At the same time, colonies may shift their activity to perennial trees in adjacent habitat patches and microhabitats as environmental conditions change throughout the season.

Among the less abundant species, *Formica rufa* (Linnaeus, 1761) and *Formica rufibarbis* (Fabricius, 1793) were found exclusively in the chestnut orchard. *F. rufa* is typically associated with forest pines, building large nests with conifer needles. This capture may reflect occasional foraging activity or the influence of nearby pine stands in the surrounding landscape. The presence of *F. rufibarbis*, a thermophilic species of open habitats, would be consistent with the sunnier structure of the chestnut orchard [66]. Overall, although chestnut orchards hosted more exclusive taxa, oak forests may function as microclimatic refugia for species associated with more stable environmental conditions under Mediterranean warming scenarios.

## 5. Conclusions

This study suggests clear differences between habitats, not only in species composition but also in their functional structure and seasonal dynamics. The chestnut orchards, as managed agroecosystems, support a higher overall ant abundance but are dominated by generalist and opportunistic guilds that thrive in disturbed environments. In contrast, the more stable, semi-natural oak forests may function as refugia for specialized guilds that depend on ecosystem complexity.

These findings have direct implications for conservation and land management. Agricultural practices in chestnut orchards may act as environmental filters, shaping the functional ant communities. The stability of species richness and functional traits in the oak forests, contrasting with the seasonal fluctuations in the chestnut orchards, suggests their role as refugia for specialist taxa, which are vulnerable to land-use change.

Finally, to capture the full scope of biodiversity, future investigations should address the limitations of this study by incorporating complementary sampling methods (e.g., manual collection or sweep netting) to better target arboreal species typically underrepresented by pitfall traps, extending sampling to a wider range of habitat types across the Montesinho Natural Park landscape, and conducting long-term studies to account for interannual variability. It would also be important to quantify the specific ecosystem services provided by these different communities, such as seed dispersal rates by the granivorous guild in the chestnut orchard and herbivore control by the predator guilds. Overall, this study highlights the need to further develop myrmecological research in Portugal, a region that remains relatively underexplored despite its potential for advancing our understanding of biodiversity and functioning of Mediterranean ecosystems.

## Figures and Tables

**Table 1:** Ant species identified in emblematic habitats in the Natural Park of Montesinho: feeding habits (No Information—NI), habitat and climatic preferences (Cold—C, Neutral—N, Warm—W and Dry—D) of the ants are indicated. Information about feeding habits, habitat and climatic preference was obtained from Arcos & García (2023) [23].

Genus	Species	Feeding Habits	Habitat Preferences	Climatic Preferences
*Aphaenogaster*	*gibbosa*	Generalist omnivore	Open Forest/Woodland	W
	*iberica*	Generalist omnivore	Generalist habitat	N/W
*Camponotus*	*aethiops*	Aphids and Nectivorous	Forest	W
	*cruentatus*	Aphids, Nectivorous and Predator	Generalist habitat	W
	*fallax*	Opportunistic and Insectivorous	Generalist habitat	W
	*micans*	Aphid, Nectivorous and Scavenger	Dry scrubland	W/D
	*piceus*	Aphids and Nectivorous	Meadow/Open Area	W
	*pilicornis*	Aphids and Nectivorous	Scrubland/Shrubland	N/C
*Cataglyphis*	*iberica*	Scavenger	Meadow/Open Area	W
*Crematogaster*	*scutellaris*	Aphid, Insectivorous and Scavenger	Forest	W/D
*Formica*	*cunicularia*	Aphid, Nectivorous, and Predator	Meadow/Open Area	W
	*decipiens*	Aphid, Nectivorous, and Predator	Edge/Transition Zone	N
	*fusca*	Aphid, Nectivorous, and Predator	Edge/Transition Zone	W
	*rufa*	Aphid and Insectivorous	Forest	N
	*rufibarbis*	Aphid, Nectivorous, and Predator	Meadow/Open Area	W
	*gerardi*	Generalist omnivore	Forest	W
*Iberoformica*	*subrufa*	Scavenger	Open Forest/Woodland	W
*Lasius*	*emarginatus*	Trophobiotics, Aphids and Scavenger	Forest	N/C
	*grandis*	Aphid, Nectivorous, and Insectivorous	Generalist habitat	N
	*niger*	Aphids and Scavenger	Forest	N/C
*Messor*	*capitatus*	Granivorous, Nectivorous and Scavenger	Scrubland/Shrubland	W/D
*Myrmica*	*aloba*	Generalist omnivore	Meadow/Open Area	N
*Pheidole*	*pallidula*	Generalist omnivore	Generalist habitat	W/D
*Plagiolepis*	*schmitzii*	Generalist omnivore	Scrubland/Shrubland	W/D
*Proformica*	*nasuta*	NI	Scrubland/Shrubland	W
*Tapinoma*	*erraticum*	Aphid, Nectivorous, and Insectivorous	Meadow/Open Area	W
*Temnothorax*	*alfacarensis*	NI	Edge/Transition Zone	W
	*tuberum* group			
*Tetramorium*	*forte*	Generalist omnivore	Meadow/Open Area	N
	*semileave* group			

## Data Availability

The dataset supporting the results of this study is available in Zenodo under the Creative Commons Attribution 4.0 licence: Lourenço-Lima, C.; Villa, M. Ant community dataset from chestnut orchards and oak forests in Northern Portugal. Zenodo. https://doi.org/10.5281/zenodo.18936619 [18936619].

## Supplementary Materials

The following supporting information can be downloaded at: https://www.mdpi.com/article/10.3390/insects17050505/s1, Figure S1: Total abundance of each ant species ranked from most to least frequent across all sampling plots and months. The red dashed line indicates the threshold (n = 40 individuals) used to distinguish ‘abundant’ (left) from ‘rare’ species (right); Table S1: Outputs for the estimated regression parameters and standard errors of the response of the ant species, richness, and Shannon diversity index to the habitat type (*Quercus rotundifolia* and *Castanea sativa*), and month (May, June, July, September, and October 2022). The structure of the model is indicated for each model.

## Abbreviations

The following abbreviations are used in this manuscript:

NPM	Natural Park of Montesinho
NMDS	Non-metric Multidimensional Scaling
PERMANOVA	Permutational Multivariate Analysis of Variance
GLMs	Generalized Linear Models
GAMs	Generalized Additive Models
PERMDISP	Permutational Analysis of Multivariate Dispersions
IPMA	Instituto Português do Mar e da Atmosfera
