# Supplementary material for: Land Use Shapes Ant Communities: Functional and Compositional Differences Between Oak Forests and Chestnut Orchards in Mediterranean Mountain Landscapes of Northern Portugal
Source: Insects. 2026 May 15;17(5):505. doi: 10.3390/insects17050505 (PMC13207051; doi:10.3390/insects17050505)
Supplement: Supplementary file 1 [file insects-17-00505-s001.zip › insects-4271862-supplementary.pdf]

Land Use Shapes Ant Communities: Functional and Compositional Differences Between Oak Forests and Chestnut Orchards in Mediterranean Mountain Landscapes of Northern Portugal

Camila Lourenço [1] Fátima Gonçalves [2], María Villa [1*]

1 CIMO, LA SusTEC, Instituto Politécnico de Bragança, Campus de Santa Apolónia, 5300-253 Bragança, Por-tugal.; camilalima@ipb.pt

2 Centre for the Research and Technology of Agro-environmental and Biological Sciences, CITAB, Inov4Agro, Universidade de Trás-os-Montes e Alto Douro, UTAD, Quinta de Prados, 5000-801 Vila Real, Portugal; mariafg@utad.pt

* Correspondence: mariavilla@ipb.pt; Tel.: +351-273303344

CLIMATE CONDITIONS IN THE SAMPLING PERIOD

To characterize the climatic conditions during the study period (May to October 2022), data from the climatic bulletins of the Portuguese Institute for Sea and Atmosphere (IPMA) for the Bragança meteorological station were used, as it is the nearest and most representative for the region.

The average monthly temperature increased from 16°C in May to a peak of 24°C in July and August, before decreasing to 18°C in September and 14°C in October. Summer heat was particularly intense, with an absolute maximum of 41.3°C recorded in July. The precipitation pattern was inversely related to temperature, with dry summer months, especially August (4.9 mm), while the autumn was significantly wetter, with October recording the highest accumulated precipitation of the sampling period, totaling 110.8 mm.

ABUNDANCE OF ANTS PER SPECIES

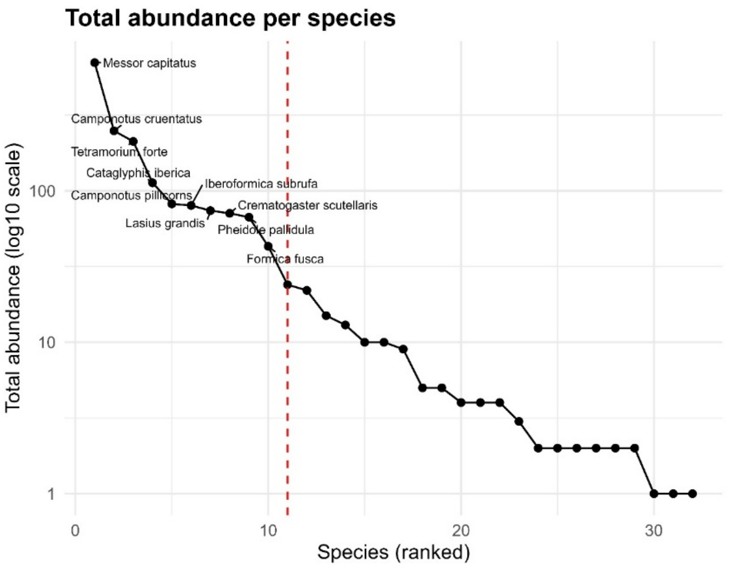

Figure S1. Total abundance of each ant species ranked from most to least frequent across all sampling plots and months. The red dashed line indicates the threshold (n = 40 individuals) used to distinguish 'abundant' (left) from 'rare' species (right).

Table S1. Outputs for the estimated regression parameters and standard errors of the response of the ant species, richness, and Shannon diversity index to the habitat type (*Quercus rotundifolia* and *Castanea sativa*), and month (May, June, July, September, and October 2022). The structure of the model is indicated for each model.

Messor capitatus				
**Generalized Linear Model: glm.nb(Abundance ~ ns(Month, df = 4))**				
	**Estimate**	**SE**	**Z value**	**p-value**
(Intercept)	0.118	0.621	0.190	0.850
ns(Month, df = 4)1	3.443	1.394	2.469	0.014
ns(Month, df = 4)2	-5.286	1.736	-3.044	0.002
ns(Month, df = 4)3	-2.505	1.636	-1.531	0.126
ns(Month, df = 4)4	-0.191	0.900	-0.212	0.832

Camponotus cruentatus				
**Generalized Linear Model: glm.nb(Abundance ~ poly(Month, 2) *Environment)**				
	**Estimate**	**SE**	**Z value**	**p-value**
(Intercept)	-1.949	0.567	-3.437	0.001
poly(Month, 2)1	28.703	8.621	3.330	0.001
poly(Month, 2)2	-31.768	7.570	-4.197	0.000
EnvironmentOak	1.907	0.624	3.054	0.002
poly(Month, 2)1:EnvironmentOak	-28.450	9.464	-3.006	0.003
poly(Month, 2)2:EnvironmentOak	16.810	8.477	1.983	0.047

Tetramorium forte				
**Generalized Linear Model: glmmTMB(Abundance ~ poly(Month, 3) + Environment, ziformula = ~1, family = nbinom1)**				
	**Estimate**	**SE**	**Z value**	**p-value**
(Intercept)	0.975	0.277	3.521	0.000
poly(Month, 3)1	5.548	3.764	1.474	0.140
poly(Month, 3)2	-2.332	2.870	-0.813	0.416
poly(Month, 3)3	-7.176	1.965	-3.652	0.000
Oak (vs. Chestnut)	-2.760	0.466	-5.925	0.000

Cataglyphis iberica				
**Generalized Linear Model: glmmTMB(Abundance ~ poly(Month, 3), ziformula = ~1, family = nbinom1)**				
	**Estimate**	**SE**	**Z value**	**p-value**
(Intercept)	-0.449	0.526	-0.854	0.393
poly(Month, 3)1	0.669	6.349	0.105	0.916
poly(Month, 3)2	-17.637	5.101	-3.458	0.001
poly(Month, 3)3	7.358	4.810	1.530	0.126

Camponotus pilicornis				
**Generalized Linear Model: glmmTMB(Abundance ~ poly(Month, 2) + Environment, ziformula = ~1, family = nbinom1)**				
	**Estimate**	**SE**	**Z value**	**p-value**
(Intercept)	-3.017	0.515	-5.859	0.000
poly(Month, 2)1	19.645	5.175	3.796	0.000
poly(Month, 2)2	-16.088	4.231	-3.802	0.000
EnvironmentOak	1.568	0.421	3.729	0.000

Iberoformica subrufa				
**Generalized Linear Model: glmmTMB(Abundance ~ poly(Month, 2) + Environment , ziformula = ~1, family = nbinom2**				
	**Estimate**	**SE**	**Z value**	**p-value**
(Intercept)	0.612	0.879	0.697	0.486
poly(Month, 2)1	-19.989	14.730	-1.357	0.175
poly(Month, 2)2	-17.213	7.045	-2.443	0.015
EnvironmentOak	-3.793	1.265	-2.997	0.003

Lasius grandis				
**Generalized Linear Model: glm.nb(Abundance ~ ns(Month, df = 4))**				
	**Estimate**	**SE**	**Z value**	**p-value**
(Intercept)	-1.681	0.739	-2.275	0.023
ns(Month, df = 4)1	2.720	1.411	1.929	0.054
ns(Month, df = 4)2	0.650	1.381	0.470	0.638
ns(Month, df = 4)3	-0.973	1.767	-0.551	0.582
ns(Month, df = 4)4	-1.165	1.137	-1.025	0.305
Oak (vs. Chestnut)	-0.095	0.603	-0.158	0.875

Crematogaster scutellaris				
**Generalized Linear Model: glmmTMB(Abundance ~ Month * Environment , ziformula = ~1, family = nbinom2)**				
	**Estimate**	**SE**	**Z value**	**p-value**
(Intercept)	3.323	0.844	3.935	0.000
Month	-0.250	0.118	-2.123	0.034
Oak (vs. Chesnut)	-5.785	2.050	-2.822	0.005
Month:Environment	0.516	0.247	2.088	0.037

Pheidole pallidula				
**Generalized Linear Model: glm.nb(Abundance ~ Month)**				
	**Estimate**	**SE**	**Z value**	**p-value**
(Intercept)	-5.352	1.073	-4.989	0.000
Month	0.510	0.128	3.976	0.000

Formica fusca				
**Generalized Linear Model: glm.nb(Abundance ~ Month+Environment)**				
	**Estimate**	**SE**	**Z value**	**p-value**
(Intercept)	-2.022	0.610	-3.316	0.001
Month	-0.006	0.164	-0.036	0.972
Oak (vs. Chesnut)	0.652	0.472	1.383	0.167

Shannon Diversity Index				
**Generalized Aditive Model: gam(Shannon ~ Environment+s(Month),by=Environment)**				
	**Estimate**	**SE**	**Z value**	**p-value**
(Intercept)	0.473	0.048	9.911	0.000
Oak (vs. Chestnut)	-0.083	0.068	-1.226	0.222
	**edf**	**Ref.df**	**F**	**p-value**
s(Month):EnvironmentChestnut	3.924	4	9.621	0.000
s(Month):EnvironmentOak	3.236	4	3.673	0.002

Richness				
**Generalized Aditive Model: gam(Riqueza ~ Environment+s(Month, by=Environment), family = nb())**				
	**Estimate**	**SE**	**Z value**	**p-value**
(Intercept)	0.520	0.113	4.607	0.000
Oak (vs. Chestnut)	-0.175	0.162	-1.081	0.280
	**edf**	**Ref.df**	**Chi.sq**	**p-value**
s(Month):EnvironmentChestnut	3.689	4	25.25	0.000
s(Month):EnvironmentOak	2.360	4	10.57	0.005
